# Animals Fed Insect-Based Diets: State-of-the-Art on Digestibility, Performance and Product Quality

**DOI:** 10.3390/ani9040170

**Published:** 2019-04-16

**Authors:** Laura Gasco, Ilaria Biasato, Sihem Dabbou, Achille Schiavone, Francesco Gai

**Affiliations:** 1 Department of Agricultural, Forest and Food Sciences, University of Turin, Largo Paolo Braccini 2, 10095 Grugliasco (TO), Italy; ilaria.biasato@unito.it; 2 Department of Veterinary Sciences, University of Turin, Largo Paolo Braccini 2, 10095 Grugliasco (TO), Italy; sihem.dabbou@unito.it (S.D.); achille.schiavone@unito.it (A.S.); 3 Institute of Science of Food Production, National Research Council, Largo Paolo Braccini 2, 10095 Grugliasco (TO), Italy; francesco.gai@ispa.cnr.it

**Keywords:** Aquaculture, Insects, Pigs, Poultry, Rabbits, Sensory

## Abstract

**Simple Summary:**

The need for new alternative nutrient sources for feed production, to face the expected future consumer demand for animal products, has led to an increase in research on the possible uses and effects of insect-derived products (meals and oils). Insects seem to be one of the most promising alternatives to other nutrient sources, such as the soybean and fish meals commonly used in feeds for livestock and aquaculture. When using alternative nutrient sources, not only do the effects on animal performance have to be evaluated, but also such parameters as nutrient digestibility and product quality as they are of primary importance for feed producers and consumers, respectively.

**Abstract:**

In 2018, the industrial compound feed production throughout the world was 1.103 metric billion tons, which was an increase of 3% compared to 2017. In order to meet the needs of the increasing population, a further increment in compound feed production is necessary. Conventional protein sources are no longer suitable to completely satisfy the increment of feed production in a sustainable way. Insects are one of the most promising options, due to their valuable nutritional features. This paper reviews the state-of-the-art of research on the use of insect meals and oils in aquatic, avian and other animal species diets, focusing mainly on the effects on digestibility, performance and product quality. In general, insect-derived product digestibility is affected by the insect species, the inclusion levels and by the process. Sometimes, the presence of chitin can lead to a decrease in nutrient digestibility. The same considerations are true for animal performance. As far as product quality is concerned, a dramatic effect of insect products has been recorded for the fatty acid profile, with a decrease in valuable n3 fatty acids. Sensory analyses have reported no or slight differences. Insect-derived products seem to be a good alternative to conventional feed sources and can make an important contribution to the sustainable development of the livestock industry.

## 1. Introduction

The increasing interest in insects as food and feeds has led to a notable development of research papers. When “Edible insects” are used as the key words in the Web of Science (WOS) database, the main results indicate that only nine papers were published in 2006, while 151 were issued in 2018, and more than 40 papers have already appeared in the first 3 months of 2019.

Although more than 2100 insect species are being eaten in various parts of the world [1], the “Yuk factor” is still strong in Europe and people are reluctant to use insect-based foods on a regular basis [2]. However, the acceptance of insects or insect-derived products in animal feed seems to be much easier [3].

By 2050, the world will host more than 9 billion people, and this will lead to food security and environmental issues. In particular, a dramatic increase in the demand for animal products is expected. In 2018, the industrial compound feed production was 1.103 metric billion tons, that is, an increase of 3% compared to 2017 [4]. Since the growing consumer demand for animal products will be met by concentrated animal feeding operations, with a further dramatic increase in the consumed feeds, great research efforts have been made to find new ingredients. Moreover, since feeds represent one of the major costs from an environmental point of view (land, energy and water uses) [5], there is a pressing need to find alternative sustainable ingredients for animal diets. The use of insects for feeds is widely recognized as one of the potential solutions, as they are rich in valuable nutrients (proteins, amino acids (AA), fat and energy, vitamins and minerals), and have a lower environmental impact than other protein sources (i.e., whey, egg protein, fishmeal) [6,7].

As stressed in the first part of the Introduction, there is an increasing interest in the use of insects as food and feed, and this, in turn, has led to an increased number of insect-related scientific publications. Therefore, the purpose of this review is to report all the current and up-to-date literature available on the use of insects as feed for monogastric animal production systems, organizing it in a single, easy-to-read document. In particular, the attention will be herein focused on the use of insect-derived products (protein meals and oils/fats) for livestock (poultry, rabbits and pigs) and aquaculture species and the related effects on digestibility, performance and product quality. Furthermore, the use of well-organized, summary tables reporting all the dietary ingredient inclusion and substitution levels will help the readers to clearly understand the topic, also providing them with a practical, useful instrument for diet formulation.

## 2. Digestibility of Insect-Derived Products in Diets

### 2.1. Fish and Shellfish

Several digestibility trials have been carried out on different aquaculture fish and shellfish species-fed feeds containing insect meals with *Tenebrio molitor* (TM) and *Hermetia illucens* (HI), which are the most frequently investigated insect species (Table 1). Trials on the use of full fat TM in gilthead seabream (*Sparus aurata*) and seabass (*Dicentrarchus labrax*) aquafeeds were carried out by Piccolo et al. [8] and Gasco et al. [9], respectively. Piccolo et al. [8] recorded lower apparent digestibility coefficients (ADC) of crude protein (CP) and ether extract (EE) for fish fed a diet containing 500 g/kg of TM than those fed low TM level (250 g/kg)- and fishmeal (FM)-based diets. Similar results were also obtained in a rainbow trout (*Oncorhynchus mykiss*) trial where the ADC of protein was significantly lower in a group fed 500 g/kg of dietary TM than in the groups fed diets with 250 g/kg of TM and FM, while the dry matter (DM), organic matter (OM) and EE ADCs were unaffected by TM meal utilization [10]. The in vivo ADC of European sea bass (*Dicentrarchus labrax*) juvenile diets with an inclusion of 25% of TM, with or without the presence of exogenous enzymes (carbohydrases and proteases), was compared to a FM-based control diet [9]. The CP ADC of the fish fed the TM diet without the presence of exogenous enzymes was significantly higher than that of the fish fed the FM-based diet [9]. On the other hand, the CP and acid detergent fiber digestibility were not improved by digestive enzyme supplementation [9]. The use of a full-fat TM as a replacement for FM has also been tested in a Pacific white shrimp (*Litopenaeus vannamei*) diet consisting of 84.5% of the reference diet and 15% of TM. The ADC values were 76.1% for CP and 66.5% for energy, while the essential amino acid (EAA) ADCs ranged from 72% to 86%, with methionine representing the first limiting AA. The authors therefore suggested that although methionine should be added as a supplement, TM can be utilized as an alternative protein source for Pacific white shrimp (*Litopenaeus vannamei*) juveniles [11]. Another insect meal that is being tested more and more frequently in feeds is HI meal, as it is probably the most promising insect for feed purposes, due to its ability to be reared on different organic substrates, thus exalting the circular economy/zero waste principle [12,13]. Fresh-water Atlantic salmon (*Salmo salar*)-fed diets containing 60% of HI, in replacement of FM, and a soybean protein concentrate, reduced the ADC of the CP, lipids and all the investigated AAs, although they remained highly digestible and comparable with the digestibility obtained for salmonids fed other alternative protein sources (poultry by-products, wheat gluten or bacterial protein meal) [14]. The use of an HI meal derived from larvae grown on seaweed, in the diets of sea-water phase Atlantic salmon (*Salmo salar*) (inclusion rates of 4.91, 9.84 and 14.75%), as partial or total replacement of FM, did not affect the ADC of the CP, lipid, AAs, or fatty acids (FA) [15]. The authors found that the apparent digestibility values of the AAs were comparable with those observed in other fish studies on salmonids (*Salmo salar*) [15,16] and European seabass (*Dicentrarchus labrax*) [17] fed HI meal and that, except for the methionine and lysine contents, they had an essential and non-essential AA profile close to that of FM. The use of high inclusion levels (40%) of HI defatted meal (CP: 55%), in substitution of FM in rainbow trout (*Oncorhynchus mykiss*) diets, was found to lead to a decrease in DM and CP ADCs, while the EE and gross energy (GE) ADCs were unaffected [18]. Dumas et al. [16] found comparable results for CP and DM in rainbow trout-fed diets including 20% of HI meal, but lower EE ADC than the ones reported by Renna et al. [18]. Chitin was hypothesized to be the cause of the low observed EE ADC [16]. Nevertheless, as higher hydroxyproline and tryptophan ADCs were found *vs* fish fed control diets containing 20% of FM, Dumas et al. [16] suggested a possible positive effect of chitin on prolidase activity, which is the specific enzyme responsible for hydrolysis and absorption of proline and hydroxyproline in the small intestine.

### 2.2. Poultry

Table 2 reports the effects on the apparent digestibility coefficients of the dietary inclusion of insect meal in avian species.

In the first available study about full-fat HI and TM digestibility in broiler chickens, De Marco et al. [19] observed similar apparent digestibility coefficients of the total tract (CTTAD) for DM, CP and GE, whereas the EE was more digestible in HI meal than in TM meal (0.99 and 0.88, respectively). On the other hand, the AA apparent ileal digestibility coefficients (AIDC) reported in their study were higher in TM meal (0.86) than in HI meal (0.68). The same authors were also the first to provide data on the apparent metabolizable energy (AME) and the nitrogen-corrected AME (AMEn) of HI and TM meals, which is useful information for formulating broiler diets (AME = 17.38 and 16.86 MJ/kg DM, respectively; AMEn = 16.60 and 16.02 MJ/kg DM, respectively). 

In order to facilitate and improve the use of insect meal in feed formulations, manufacturers have started to produce defatted larva meals. Defatted insect meals can in fact be an optimal way of providing high protein insect meals and lipid or fat by-products, which, in turn, have a great potential for alternative purposes [20]. Following the same approach as De Marco et al. [19], Schiavone et al. [21] provided new information concerning CTTAD, AME, AMEn, and AIDC of a partially and a highly defatted HI larva meal (HIp and HIh, respectively) for broiler chickens as replacement of 250 g/kg of the basal diet. They observed that both the EE and the GE ADCs were higher in HIp (0.98 and 0.61 for the EE and GE, respectively) than in HIh (0.93 and 0.50, respectively). However, no significant difference was found for the CTTAD of CP. The same authors also showed that the animals were able to utilize the AA of both the partially and the highly defatted HI meals (AIDC of 77–80% for HIp and HIh, respectively), with AIDC overall not being affected by the defatting process (except for glutamic acid, proline and serine). Furthermore, as a consequence of the different defatting processes, the HIp meal showed higher levels of AME and AMEn (16.25 and 14.87 MJ/kg DM, respectively) than the HIh meal (11.55 and 9.87 MJ/kg DM, respectively) [21].

From a general point of view, De Marco et al. [19] and Schiavone et al. [21] speculated that chitin can negatively affect the digestibility of protein and reduce the general digestion of DM. Nevertheless, these authors pointed out that TM and full-fat or partially defatted HI meals can be an excellent source of energy and digestible AA for broiler chickens, regardless of the presence of chitin. 

As far as the different defatting processes adopted for insect meal production are concerned, Uushona [22] was the first to investigate the combined effects of heat and the defatting method on HI digestibility in broiler chickens. The author observed that diets containing defatted HI pre-pupa meal dried at 65 °C were characterized by higher nutrient CTTAD and AME than those containing full-fat HI pre-pupa meal dried at 65 °C. Cockcroft [23] also focused attention on two different defatted HI larva meal diets (namely dry rendered (DR) and extruded) and compared their effects with those of a full-fat HI and a control diet. They observed that DR diets showed the higher CTTAD for CP, EE, ash, and crude fiber.

As far as various dietary inclusion levels are concerned, Cullere et al. [24] reported higher CTTAD of EE in growing broiler quails fed a 15% level of defatted HI larva meal inclusion (89.6%) than those fed 10% (82.5%). Woods et al. [25], in a trial with HI reared on layer mash and an equal mixture of layer mash and fish offal, recently observed a higher AME for larva-fed quails. Quails fed an equal mixture of layer mash and fish offal showed higher CTTAD for DM and OM. However, no significant differences were observed for the CTTAD of DM, OM or CP. On the contrary, high inclusion levels of TM larva meal (29.6%) in the diets of broiler chickens have been reported to lower the AIDC of DM, CP and OM [26]. Bovera et al. [27] also observed a decrease in the AIDC of DM, OM and CP in HI-fed laying hens as the percentage of insects increased, with the negative effects being particularly evident when HI larva meal was included at 14.6% in their diets. Analogous findings, in terms of nutrient digestibility, were also obtained by Cutrignelli et al. [28] for laying hens fed diets with high inclusion levels (17%) of HI larva meal.

Hwangbo et al. [29] instead tested a diet containing 300 g/kg dried house fly (*Musca domestica*, MD) larva meal on broiler chickens and observed a very high CTTAD of CP (0.98) in comparison with a soybean meal-ground yellow corn-soybean oil-based diet.

Gariglio et al. [30] showed that in broiler ducks fed 3, 6 and 9% of HI larva meal, in substitution of gluten meal, the CTTAD of CP decreased during the starter period and the lowest CP value was registered for the inclusion of 9% HI. However, the CTTAD of EE increased with increasing HI inclusion levels during the grower and finisher periods [30].

Broilers fed diets with TM oil inclusion, in substitution with soybean oil, showed an increase in the EE of CTTAD [31]. The same authors, in a second trial in which both TM and *Zophobas morio* oils were used, reported no differences in the ideal digestibility of CP, EE or AMEn [31].

### 2.3. Other Animal Species

The effects of dietary insect meal inclusion on the nutrient digestibility of pigs and rabbits are reported in Table 3.

#### 2.3.1. Pigs

The digestibility trials conducted so far have led to heterogeneous results, depending on the insect species and life stages, as well as on the dietary inclusion levels. Low inclusion levels of HI pre-pupa and larva meal (4–8% [32] and 5-10% [33], respectively) in weaned piglet diets have been reported to have no effect on nutrient digestibility, while TM larva meal utilization (low inclusion rates of 1.5–6%) determines a linear improvement in DM and CP digestibility [34]. Yoo et al. [35] also observed a higher apparent ileal digestibility (AID) of lysine, histidine, arginine, and cysteine in growing pigs fed TM larva meal-based diets (low inclusion level of 9.95%) than in pigs fed other protein source diets, as well as a greater standardized ileal digestibility (SID) of GE and cysteine. On the other hand, Newton et al. [36] observed lower DM and higher EE digestibility in piglets fed a high inclusion level (33%) of dried HI larva meal than those fed soybean meal (SBM). Increasing levels (from 5 to 20%) of a rumen content-MD larva meal mixture in the diets of early weaned piglets has also been reported to not affect nitrogen digestibility [37].

#### 2.3.2. Rabbits

Very few researches have concerned the digestibility of insect-derived products in rabbits, with contrasting results being reported, in particular when different dietary inclusion levels have been considered. Dietary HI and TM larva fat utilization at low inclusion rates (0.75–1.5%) has been reported not to affect the nutrient digestibility of weaned rabbits [38]. However, the authors also found that higher inclusion levels (3–6%) of HI larva fat led to lower DM, OM, EE and GE digestibility than when extruded linseed was used [39]. However, the same authors also observed that increasing the dietary level of HI fat determined a significant increase and decrease, respectively, in EE and cellulose digestibility [39].

## 3. Performance

### 3.1. Fish and Shellfish

In a similar way to what is reported in the nutrient digestibility section, the most frequently investigated insect species in fish and shellfish growth trials have been found to be TM and HI (Table 4). As far as the dietary utilization of TM meal is concerned, some of the performed nutritional trials were carried out on juvenile fish with initial body weights ranging from 3 to 20 g, and the tested TM was a full-fat meal with a CP content ranging from 52 to 56% on a DM basis [9,40]. In juvenile rockfish (*Sebastes schlegeli*) fed experimental diets containing TM levels up to 32%, the fish weight gain (WG) and specific growth rate (SGR) increased with increasing TM dietary inclusions up to 16%, while they then tended to decrease for higher levels in comparison with those fed FM-based diet [41]. In another trial, performed on mandarin fish (*Siniperca scherzeri*) juveniles fed full-fat TM at inclusion levels up to 30%, the results revealed an increase in fish growth rates and in the efficiency of nutrient utilization for up to 20% of TM inclusion, while a decline was observed when the dietary TM level increased from 20 to 30% compared to those fed the FM-based diet [40]. Similar conclusions were found by Gasco et al. [9] for European seabass (*Dicentrarchus labrax*) juveniles fed a diet with 25 and 50% TM inclusion levels. In the same study, the final body weight, WG, SGR, and feeding rate were comparable in the 25% TM group, with respect to the control FM based diet, while these parameters worsened for the highest inclusion level (50%) [9]. The TM meal utilization in shellfish, particularly in Pacific white shrimp (*Litopenaeus vannamei*) diets, was also investigated by Panini et al. [11], who found that WG, SGR, feed intake (FI), feed conversion ratio (FCR), survival, and protein retention were not affected when FM was partially or totally replaced in diets containing up to 305 g/kg of a full fat TM meal. However, few studies are available on the use of a defatted TM meal. On juvenile pearl gentian grouper, Song et al. [42] tested a TM meal with a 65% CP content in a feeding trial that lasted 50 days. The fish were fed up to 31.5 % of graded levels of TM meal and the authors found a lower final weight and WG rate in the control fish and in the 6.25% inclusion level groups, while the FCR of the fish fed 12.5 and 18.75% TM diets showed no significant difference compared to the control diet. In order to obtain a maximum WG rate and according to a broken-line model, authors recommended a TM inclusion level of 4.92%, corresponding to a FM substitution of 12.3% [42].

As far as the utilization of HI meal is concerned, most of the fish feeding trials that have been performed have shown that FM replacement is feasible at low and moderate inclusion levels, without any negative effects on the growth performance or FCR. The survival, growth performance and FCR of salmonids, particularly in rainbow trout, were not affected in fish-fed diets in which 13%, [16], 46% [43], or 50% [18] of FM have been replaced with a defatted HI meal for fish with a mean initial body weight of 46, 67 or 179 g, respectively. Similar conclusions on fish growth were obtained by Sealey et al. [44] in a trial carried out on rainbow trout, where 25 and 50% of FM were substituted with fish offal-enriched HI pre-pupae. Belghit et al. [15] also observed that a partial or total replacement of the diets of sea-water phase Atlantic salmon (*Salmo salar*) containing 100 g/kg FM with insect meal did not affect the FI, daily growth increase or FCR in fish with an initial body weight of 1400 g. Similar results, in terms of growth performance and feed utilization, were found by Zhou et al. [45] and Magalhães et al. [17]. These authors concluded that up to 14% and 19.5% of HI meal can be included in diets of Jian carp and juvenile European seabass (*Dicentrarchus labrax*), respectively. HI meal has also been tested in diets for the juveniles of clownfish (*Amphiprion ocellaris*), an ornamental marine fish species, which were fed graded levels of a defatted HI meal in partial or total substitution of FM over a 106-day experimental period [46].

In the same study, biometric results did not reveal any differences in terms of survival and growth, and these findings were confirmed by means of Real-Time PCR analyses on the genes involved in fish growth [46]. Moreover, as far as shellfish nutritional trials are concerned, only one study is available on the replacement of FM with HI meal in the diets of Pacific white shrimp (*Litopenaeus vannamei*) juveniles [47]. These authors found that if the replacement was limited to 25% of the diet, most of the growth responses, such as the WG, SGR, and FCR parameters, were not affected by the dietary HI meal content.

### 3.2. Poultry

Among the various insect species (Table 5), HI larva meal is the most widely used, and it is also the one that has been studied the longest as a protein source for poultry feeding [48]. This first research showed that chicks fed a diet containing dried HI larvae (as a substitute for SBM) gained weight at a rate of 96% (even though non-significant) compared to that of birds fed the control diet, but they only consumed 93% (significant) as much feed. The majority of studies on broiler chickens and quails fed HI larva meal, compared to broilers and quails fed conventional protein sources, have revealed that there is no influence on the growth performance of the birds [22,24,49,50], with broiler quails being reported to prefer diets including HI larva meal [24]. In one of the most recent studies performed on broiler chickens, Dabbou et al. [51] observed that the inclusion up to 10% of HI larva meal in partial substitution of SBM improved the final live weight (LW) and the FI during the starter period, even though the FCR of the growing and finisher periods resulted in impaired HI-fed birds (in particular those fed a 15% inclusion level) [51]. On the other hand, Barbary partridges (*Alectoris barbara*) fed dietary inclusions of 100 and 190 g/kg of HI larva meal in substitution of 25 and 50% SBM, respectively, resulted in a higher LW and better FCR than the control group [52].

As far as laying hens are concerned, no significant differences have been observed for diets containing partially defatted HI larva meal of dried HI larvae as a partial or complete replacement of soybean cake [53]. Al Qazzaz et al. [54] reported an unaffected or improved growth performance and productivity in laying hens fed diets supplemented with 1% or 5% of HI larva meal. On the contrary, laying hens fed diets in which the SBM was completely replaced by HI larva meal showed a more favorable FCR than birds fed the control diet, but a lower laying percentage, FI, average egg weight, and egg mass [55]. Mwaniki et al. [56] showed that the inclusion up to 7.5% defatted HI larva meal in a corn–SBM diet fed to pullets (19 to 27 weeks of age) increased FI and FCR. Bovera et al. [27] observed no change in the egg weight, FI or FCR in HI-fed laying hens, although the egg mass and the laying percentage were positively affected by the lowest inclusion levels of HI larva meal (7.3%). Hens fed diets containing HI have also been reported to show higher egg production and weight than those fed a control diet [57].

Widjastuti et al. [58] reported that the substitution of FM with HI meal in the diets of female Japanese quails had better effects on the FI, FCR and egg weight, while the egg production was unaffected by dietary IM inclusion.

In a recent study performed by Gariglio et al. [30], broiler ducks fed with up to 9% of HI larva meal, in substitution of gluten meal, did not show any difference in the FI or FCR during the different feeding phases.

As far as other insect species with great potential as feeds are concerned, the introduction of TM meal into broiler and free-range chicken diets, respectively, has been reported not to influence the growth performance of the animals [59,60]. On the contrary, in female and male broiler chickens fed increasing levels of full-fat TM meal inclusion (50, 100 and 150 g/kg) as partial replacement of SBM, corn gluten meal and soybean oil, Biasato et al. [61,62] showed an improvement in LW and FI, but the feed efficiency was observed to be partially impaired. The FCR of broiler chickens fed a corn-SBM-based diet in which TM larva meal was included at 29.65% as total replacement of SBM was instead positively affected by dietary insect meal inclusion [26,63]. Khan et al. [64] showed reduced feed consumption and FCR, as well as increased BWG, in broiler chickens fed diets in which TM larva meal was used to completely replace SBM. In the same context, Ballitoc and Sun [65] pointed out that an inclusion level of 10 g/kg TM in broiler chicken diets had a great impact on the growth performance of the animals, in terms of improved final LW, FI and FCR.

Regarding the use of MD in poultry diets, Adeniji [66] did not observe any effects on the performance of broiler chickens when groundnut cake was substituted with MD meal. On the contrary, Téguia et al. [67] showed that replacing fish meal with MD in the starter and grower-finisher diets led to a higher LW, without affecting the FCR. Similarly, Hwangbo et al. [29] showed that the body weight gain (BWG) was significantly higher in birds receiving 10 or 15% maggot supplementation than in the control, while the FCR was not affected by dietary insect meal inclusion. However, when Okah and Onwujiariri [68] replaced FM with maggot meal, they instead showed that the broiler chickens fed maggot meal-based diets had superior FCR than those fed the control diet.

Only two researchers have studied the effects of MD in laying hens [69,70]. MD maggot meal has been reported to have been used to replace 50% of FM in a cassava-based diet without any adverse effects on FI, FCR, egg production, or shell strength. However, 100% replacement was deleterious to hen-egg production [69]. Finally, Dankwa et al. [70] showed an improvement in the clutch size, egg weight, number of eggs hatched, and chick weight of birds supplemented with 30 and 50 g of live MD.

The use of insect oils/fats in poultry diets, in substitution of conventional lipid sources, has led to controversial results as far as the performance parameters are concerned. For instance, no differences were observed by Schiavone et al. [71], who fed broiler diets containing 3.43 or 6.87% of HI fat in partial or total substitution of soybean oil. On the other hand, Kierończyk et al. [31] in a first trial reported a decrease in FI and FCR, but no effect on BWG, when broilers were fed diets containing TM oil in substitution of soybean oil. Nevertheless, in a second trial, the same authors used TM and *Zophobas morio* oils and reported no effects on the performance parameters when considering the whole rearing period (28 days of trial), but with differences in BWG between treatments for intermediate periods (14–21 days and 21–28 days) [31].

### 3.3. Other Animal Species

#### 3.3.1. Pigs

Similarly to what has been observed for nutrient digestibility, the growth performance of pigs fed diets containing insect meal may vary remarkably (Table 6). However, these differences seem to be related to the insect species rather than to the dietary inclusion levels. The introduction of HI meal in weaned piglet diets has been reported to not influence the growth performance of the animals [32,33,72,73], even though increasing FI can be observed for increasing levels of HI meal inclusion [33]. On the other hand, TM utilization in the diets of weaning piglets has led to improved growth performance [34,74]. Similar findings have also been reported for MD (unaffected growth [75,76,77] or improved WG of the animals [78]) and *Bombyx morii* (improved WG [79]). Only Adeniji [37] observed lower FI in early weaned piglets fed diets containing MD larva meal than in piglets fed wheat offal-based diets.

#### 3.3.2. Rabbits

Researches performed on the inclusion of insect meal in rabbit diets are extremely scarce. Feeding rabbits with diets containing BM meal as the total replacement of SBM had no effects on growth performance [80]. Similarly, dietary insect fat inclusion (HI [38,39] and TM [38]) has been reported to not influence the growth performance of weaned rabbits.

## 4. Product Quality

### 4.1. Fish and Shellfish

The effects of IM utilization on the morphometric and slaughter traits, proximate composition and quality parameters of aquaculture products have been investigated through the testing of different experimental diets containing HI and TM meal (Table 7). The obtained results about the morphometric and the slaughter traits appear to be quite heterogeneous. Indeed, several studies reported no significant effects related to insect meal utilization in TM-fed blackspot seabream [81] and rainbow trout (*Oncorhynchus mykiss*) [82], or in HI-fed rainbow trout [18] and Atlantic salmon (*Salmo salar*) [15]. On the other hand, other authors observed that both the low (10.5%) and high (from 18.75 to 60%) levels of insect meal inclusion (TM [8] and HI [14,45,83,84]) in the diets of carp [45,83], gilthead seabream [8] and Atlantic salmon [14,84] had significant effects on the morphometric and slaughter traits. In particular, higher hepatosomatic indexes [14,45,84] and viscerosomatic indexes (VSI) [14,84] were identified in HI-fed Atlantic salmon [14,84] and carp [45] than those fed control diets. However, lower VSI [8,83] and a lower intraperitoneal fat index [83], as well as a lower dressed yield [8], were instead observed in HI-fed carp [83] and TM-fed gilthead seabream [8] than in fish fed control diets.

With regards to the proximate composition, high dietary inclusion levels (up to 50%) of TM larva meal did not lead to any changes in the moisture, protein or ash content in shrimp muscle [85] and rainbow trout *(Oncorhynchus mykiss)* fillets (raw and cooked) [82]. However, increased DM and EE contents of trout dorsal fillets were observed by Renna et al. [18] in fish fed the highest level (40%) of HI larva meal inclusion in their diets when compared to 20% HI- and FM-fed groups. Furthermore, Zhou et al. [45] reported no significant effects related to dietary HI larva meal utilization on the whole body composition of carp. Similarly, Belghit et al. [14] observed no changes in the whole body composition of HI-fed Atlantic salmon.

Regarding the effect of insect meals on product quality parameters, dietary TM larva meal utilization has been reported to not influence the water holding capacity or texture characteristics of fillets obtained from blackspot seabream [81], gilthead seabream [8] and rainbow trout [82]. With regard to the fish color, a higher redness index (a*) and increased yellowness (b*) were detected in the skin ventral region and fillet epaxial region, respectively, of blackspot seabream fed the maximum dietary inclusion level (40%) of TM larva meal [81]. Mancini et al. [86] instead highlighted decreased fillet yellowness in rainbow trout *(Oncorhynchus mykiss)* fed the highest inclusion level (40%) of HI larva meal in their diets.

Insect FA profiles may vary greatly according to the insect species and the substrates used for their rearing [12,13,87], which can in turn influence the fish product FA. Insect larva lipid profiles are rich in saturated fatty acids (SFA) and poor in polyunsaturated fatty acids (PUFA), the most important ones for human health being eicosapentaenoic acid (EPA) and docosahexaenoic acid (DHA). As a consequence of the high content of SFA that characterizes HI larvae, freshwater fish fed increasing levels of HI meal showed increased contents of SFA (mostly lauric acid, C12:0) and decreased contents of valuable PUFA (both n-3 and n-6) [18,45,86,88]. However, different results have been reported for HI-fed Atlantic salmon. Increasing levels of HI meal inclusion in their diets in fact led to either a decrease [84] or an increase [15] in the n-3/n-6 ratio, as well as an increase in the EPA and DHA contents [15]. TM larvae are characterized by high oleic, linoleic and palmitic acid contents [87]. Therefore, fish-fed diets with high levels of TM meal showed increased n-6 PUFA contents, to the detriment of the n-3 PUFA contents, with a consequent reduction in the Σn-3/Σn-6 FA ratio and a worsening of the atherogenicity and thrombogenicity indexes [10,81,82].

Since insect meal utilization in fish diets may lead to remarkable changes in the FA composition of the fillets, the sensory properties of fish products may also vary (in particular the aroma and flavor, which are closely related to the dietary lipid-volatile components) [89]. The training of panelists may significantly affect their capability of perceiving sensory differences, even though the so-far performed panel tests have led to similar results between trained and untrained panelists. The first available data revealed unaffected sensory parameters (perceived by both trained and untrained panelists) for rainbow trout [44] and Atlantic salmon [84] fed HI pre-pupa and larva meals, respectively. Untrained panelists also identified no significant differences in terms of taste and odor in HI-fed rainbow trout, while a darker fillet color was perceived in fish fed insect meal than in the fish of the control diet [43]. On the contrary, small to significant changes in the odor, flavor, color and texture characteristics were identified by trained panelists for rainbow trout [89] and Atlantic salmon [15] fed HI larva meal. Moreover, the fillets from HI-fed rainbow trout (inclusion levels ranging from 20 to 40%) were characterized by the onset of a dominance of a metallic flavor [89], whereas a prominent rancid odor was observed for baked fillets of Atlantic salmon fed the highest inclusion level (100%) of HI larva meal in their diets [15].

Unlike fish, the effects of dietary IM inclusion on the product quality of shellfish (in particular shrimps) have warranted very limited attention. Only Panini et al. [85] reported that dietary TM larva meal inclusion did not affect the muscle quality of Pacific white shrimp (*Litopenaeus vannamei*), even though high inclusion levels (above the 15%) increased the lipid muscle contents and decreased the PUFA muscle contents. The maximum whole-body lipid content was also observed in shrimp fed a 15% inclusion level of HI larva meal in their diets [47].

### 4.2. Poultry

The effects of dietary insect meal inclusion on the carcass characteristics and meat quality parameters of poultry products (Table 8) have been investigated by testing different inclusion levels of HI, TM and MD meals.The carcass traits of broiler chickens and quails were unaffected by dietary HI, MD, and TM larva meal inclusion [24,26,49,50,62,67,90]. However, Schiavone et al. [91] showed that an inclusion of up to 10% of HI larva meal, in partial substitution of SBM, improved the live and carcass weights of broiler chickens. Loponte et al. [52] reported greater carcass weights in Barbary partridges (*Alectoris barbara*) fed HI and TM-based diets as a partial replacement (25 % or 50%) of SBM than in the control group. Ballitoc and Sun [65] also found improved slaughter yields, dressed carcass and eviscerated weights in broiler chickens fed TM diets at a 2% inclusion level. Similarly, Biasato et al. [61] evaluated the effects of a partial replacement of SBM, corn gluten meal and soybean oil with TM larva meal on the carcass characteristics of female broiler chickens and found an increase in the carcass weight, abdominal fat weight and abdominal fat percentage with increasing levels of TM meal utilization (from 5 to 15%). Pieterse et al. [92] similarly demonstrated that broilers that received 10% MD larva meal had higher live and carcass weights, as well as higher breast and thigh yields than those of the control diet. Téguia et al. [67] did not observe any significant difference for different dietary inclusion levels of MD and the control group for the hot carcass yield and proportion of different parts of the carcass, but instead observed higher proportions of liver and gizzard.

The effect of insect meals on meat quality parameters and chemical composition have partially been studied. Studies carried out on broiler chickens [26,49,90,93] fed different HI and TM meal inclusion levels reported no significant differences for the color of broiler meat. On the other hand, Cullere et al. [24], Schiavone et al. [91] and Pieterse et al. [92] showed that HI and MD larva meals affected the meat color of broiler chickens and broiler quails, respectively. Secci et al. [94] did not observe any effect on the color of raw meat of Barbary partridges fed HI and TM meal, but an increase in the yellowness index of the cooked meat was observed.

The TM-based diets may affect the lipid content of broiler chickens when high TM larva meal levels are included in their diets [65]. However, dietary HI and TM inclusion has not been reported to affect the chemical composition of the meat of broiler quails [95], cooked chicken meat [90] or that of Barbary partridges [94]. Schiavone et al. [91] instead observed a progressive increase in crude protein and a decrease in the moisture content in the breast meat of broilers fed 5, 10 and 15% inclusion of HI larva meal in the diets.

With regards to the effect of insect meals on poultry meat FA profile, as a result of the high SFA content of HI, quails fed increasing levels of HI larva meal showed increased and decreased contents of SFA (mostly lauric acid, C12:0) and valuable PUFA (both n-3 and n-6), respectively [95]. In a similar context, Schiavone et al. [91] showed a higher SFA and monounsaturated fatty acid (MUFA) and a lower PUFA rate in broiler chickens fed different inclusion levels of HI larva meal. The same results were obtained by Secci et al. [94], who observed that Barbary partridges fed 25 or 50% of HI and TM diets were characterized by higher rates of SFA and MUFA, as well as a lower PUFA content. Feeding broilers a diet containing full-fat TM meal resulted in an increase in C12:0 and C14:0, but no differences were reported as far as quality indexes, such as the PUFA n6/n3 ratio or atherogenicety and thrombogeniticy indexes, are concerned [96].

Little information is available on the effect of insect meals on meat sensory properties. An unaffected meat sensory profile (odor, flavor, aroma, texture, juiciness, and tenderness) was observed in broiler quails [95] and chickens [50,90] fed HI larva meals. However, Altmann et al. [93] instead showed that the breast meat of broilers fed diets containing HI meal had a more intense flavor that decreased over storage time. In a study by Pieterse et al. [90], broiler chickens that were fed HI larvae produced breast meat that had a higher metallic aroma and aftertaste, and more sustained juiciness values than those fed soya bean- and fishmeal-based diets. Khan et al. [64] reported that various IM products did not affect the meat taste or the flavor, but the tenderness and the juiciness were higher in a TM-fed group than in the control and the other diets.

Al-Qazzaz et al. [54] observed a significant improvement in the appearance, texture, taste, and odor of eggs from laying hens for increasing levels of HI larva meal in the feed compared to those fed a basal diet. 

Regarding the effect of insect meals on egg quality characteristics, Mwaniki et al. [56] reported that including up to 7.5% of defatted HI larva meal in a corn–SBM diet for pullets (19 to 27 weeks of age) led to an increase in the intensity of the yolk color, as well as an increase in the egg shell-breaking strength and thickness. The same results were obtained by Secci et al. [97], who recently tested the effects of the total replacement of SBM with HI larva meal in laying hen diets (Lohmann Brown Classic) and they have reported a higher proportion of yolk in the eggs, as well as higher amounts of γ-tocopherol, lutein, β-carotene and total carotenoids in HI-fed birds. Ruhnke et al. [98] instead showed a decrease in egg weight, shell weight and thickness, as well as a decrease in the intensity of the yolk color in free-range laying hens fed HI larva meal. 

A limited number of studies evaluating the effect of insect fats in poultry diet has so far been conducted. Schiavone et al. [99], after feeding broiler diets containing 3.45 or 6.9% of HI fat in partial or total substitution on soybean oil, reported no changes in the carcass characteristics or meat quality parameters, but an increased SFA, to the detriment of the PUFA fractions. Kierończyk et al. [31] showed that TM addition had a positive effect on the PUFA, MUFA and SFA contents. The authors observed that the AI and TI values in the breast of broiler chickens were both reduced by dietary TM oil inclusion.

### 4.3. Other Animal Species

#### 4.3.1. Pigs

Bayadina and Inkina [75] were the first to investigate the meat quality of young pigs fed MD larva meal (Table 9). They found that dietary insect meal inclusion resulted in more carcass meat with good organoleptic properties. Higher slaughter weights and fat measurements were also reported for weaner pigs fed MD larva meal-based diets instead of fishmeal [77].

#### 4.3.2. Rabbits

Dalle Zotte et al. [100] recently focused attention on the meat quality traits of insect fat-fed weaned rabbits. The first result that deserves mentioning is that diets with HI larva fat reduced the intramuscular fatty acid (FA) content, but increased the 12:0 and 14:0 in meat, compared to extruded linseed-based diets. Furthermore, meat from HI-fed rabbits showed a worse atherogenic index, thrombogenic index and n-3/n-6 ratio than those of linseed-fed animals. Based on these findings, the authors concluded that the overall lipid profiles of the meat from HI-fed rabbits were not as healthy as those of linseed-fed animals [101]. On the other hand, rabbits fed diets with grass hopper meal (inclusion level of 2.5%), aimed at substituting 50% of FM, had carcasses that showed significant differences between treatments as far as the slaughter weight, carcass weight, dressing percentage, skin pelt, tail, feet and abdominal fat are concerned, with the slaughter weight and carcass weight being better in rabbits fed grass hopper meal than those fed FM [101].

## 5. Conclusions

With the world’s population continuously increasing and the growth in the demand for animal products, insect-derived products show a great potential for animal nutrition to overcome nutrient and environmental issues.

Research interest in this topic is growing, and this has led to an increase in the number of published data. The available scientific literature demonstrates that insect-derived product digestibility is impacted by the insect species, the inclusion levels and by the process (drying, defattening). Generally speaking, high digestibility values have been recorded. Sometimes, the presence of chitin can lead to a decrease in nutrient digestibility. The same considerations are true for animal performance. As far as product quality is concerned, although the effects of chitin on the proximate composition are not consistent, a dramatic effect of insect products has been recorded for the fatty acid profile, together with a decrease in the valuable n3 fatty acids and a general worsening of the nutritive value. The sensory analyses that have been performed report no or only slight differences against the product obtained using conventional nutrient sources, especially when untrained panelists are involved. Other insect-derived products, such as fat/oil or chitin, have shown a great potential, not only as a source of nutrients, but also as an immunostimulant and modulator of the animal microbiota, thus opening the way to new uses and possibilities.

The current European regulations allow insect meal from seven insect species to be used, but only for aquafeeds, while their use for pigs and poultry is still under discussion. Future researches are needed to continue to provide legislators with new data in order to support the decision making process and to obtain approval for pig and poultry feeds.

## Figures and Tables

**Table 1:** Insect meal inclusion in fish and shellfish diets and related effects on apparent digestibility coefficients of aquatic species in comparison with the control diet used in the different trials.

Aquatic Species	Insect Species	Insect Form	% Insect Inclusion	Days of Feeding	Impact on Nutrient Digestibility	Reference
Atlantic salmon	Partially defatted HI	Larva meal	4.91%, 9.84% and 14.75%	114	No effect on the ADC of the CP, CL, AA and FA.	[15]
	Partially defatted HI	Larva meal	60%	56	The ADC of the CP, CL and all AA significantly reduced.	[14]
European seabass	Defatted HI	Pre-pupa meal	6.5%, 13% and 19.5%	25	The ADC of the DM and OM, CP, EE and energy were unaffected by the diet composition. No differences were observed for the ADC of the AAs.	[17]
	Full-fat TM	Larva meal	25%	21	The CP ADC was significantly higher than the control diet. The supplementation of exogenous digestive enzymes did not improve the protein and ADF digestibility.	[9]
Gilthead seabream	Full-fat TM	Larva meal	25% and 50%	21	The estimated ADCs of the CP and EE of the diets were lower in the group fed the 50% of TM inclusion.	[8]
Pacific white shrimp	Full-fat TM	Larva meal	15%	NS	The ADC of the DM and energy showed low values. The essential AAs ADC values ranged from 72.86% to 86.41%. Methionine was the first limiting amino acid in TM.	[11]
Rainbow trout	Partially defatted HI	Larva meal	25% and 50%	21	No differences for the ADC of the EE and GE, while ADC of DM and CP were higher in the 25% compared to the 50% HI group.	[18]
	Partially defatted HI	Larva meal	20%	NS	No differences were observed for the ADC of the most nutrients except for the CL, hydroxyproline and tryptophan. The ADC of these AAs increased in trout fed HI meal, while CL decreased.	[16]
	Full-fat TM	Larva meal	25% and 50%	21	The ADC of the CP was significantly lower in the TM50 group than the other groups, while the ADC of the DM, OM and EE wasunaffected by treatment.	[10]

Note: HI: *Hemetia illucens*; TM: *Tenebrio molitor*; AA: Amino Acid; ADC: apparent digestibility coefficient; ADF: acid detergent fiber; CL: crude lipid; CP: crude protein; DM: dry matter; OM: organic matter; EE: ether extract; FA: Fatty acids; GE: Gross energy; NS: not specified.

**Table 2:** Dietary insect meal inclusion in avian diets and related effects on apparent digestibility coefficients in comparison with the control diet used in the different trials.

Avian Specie	Insect Species	Insect Form	% Insect Inclusion	Days of Feeding	Impact on Nutrient Digestibility	Reference
Broiler chickens	Full-fat TM and HI	Larva meal	25%	9	No influence on the CTTAD of the nutrients, except for the CTTAD of the EE: HI more digestible than TM. Higher AIDC in the TM group than in the HI.	[19]
	HI dried (100 °C) HI dried (65 °C) Defatted HI dried (65 °C)	Pre-pupa meal	50% 50% 40%	7	Higher CTTAD values for the nutrients in the defatted HI pre-pupae meal dried at 65 °C diet than the control and the diets with HI pre-pupae meals dried at 65 °C.	[22]
	Partially defatted HI Highly defatted HI	Larva meal	25%	9	Influence on the CTTAD of the EE and GE, which were more digestible in the HI partially defatted group than the HI totally defatted. No effect on the AA digestibility except for the glutamic acid, proline and serine that were more digestible in the HI highly defatted.	[21]
	Dry-rendered HI Extruded HI Full-fat HI	Larva meal	50%	5	Higher CTTAD of the CP, EE, ash and CF in the dry-rendered larvae diet than the other groups.	[23]
	Full fat TM		29.65%	32	Decrease in the ADC of the DM, CP and OM.	[26]
	TM	Oil	5%	28	Increase in the CTTAD of the EE.	[31]
	MD	Larva meal	30%	7	Increase in the ADC of the CP and AA.	[29]
Broiler quails	Defatted HI	Larva meal	10% and 15%	11	Higher CTTAD of EE in the 10% group than the other groups. No significant effect on the CTTAD of the DM, OM and CP.	[24]
	HI1 (reared on layer mash) HI2 (reared on 50:50 layer mash and fish offal)	Larva meal	10%	15	Higher AME for the HI-fed quails than the control diet. HI2: higher CTTAD for the DM and OM than the HI1.	[25]
Broiler ducks	Defatted HI	Larva meal	3%, 6% and 9%	47	Lower CTTAD of the CP during the starter period with the inclusion of 9% HI than the other diets. Higher CTTAD of the EE during the grower and finisher periods than the control diet.	[30]
Laying hens	Partiallydefatted HI	Larva meal	7.3% and 14.6%	140	Decrease in the AIDC of the DM, OM and CP.	[27]
	Highly defatted HI	Larva meal	17%	147	Decrease in the AIDC of the DM, OM and CP.	[28]

Note: HI: *Hemetia illucens*; TM: *Tenebrio molitor*; MD: *Musca domestica*; CTTAD: apparent digestibility coefficients of the total tract; AIDC: apparent ileal digestibility coefficients; ADC: apparent digestibility coefficient; DM: dry matter; OM: organic matter; CP: crude protein; EE: ether extract; CF: crude fiber.

**Table 3:** Effects of dietary insect product inclusion on nutrient digestibility of pigs and rabbits in comparison with the control diet used in the different trials.

Animal Species	Insect Species	% Substitution (Ingredient)	% Insect Inclusion	Days of Feeding	Impact on Nutrient Digestibility	Reference
Barrow pigs	Dried HI larva meal	100% (SBM)	33%	10	Lower DM and higher EE digestibility than the control diet.	[36]
Early weaned piglets	Dried MD larva meal in association with rumen content (mixture)	25%, 50%, 75% and 100% (wheat offal)	5%, 10%, 15% and 20%	119	No effects.	[37]
Weaning pigs	Dried full-fat TM larva meal	5%, 10%, 15% and 20% (SBM)	1.5%, 3.0%, 4.5% and 6.0%	35	Linear improvement in the DM and CP digestibility for increasing levels of dried mealworm in the diets.	[34]
	Full-fat and defatted pre-pupa meals	50% and 100% (full-fat HI pre-pupa meal replacing toasted soybeans); 70% (defatted HI prepupa meal replacing toasted soybeans)	4-8% (full-fat HI prepupa meal); 5.4% (defatted HI prepupa meal)	15	No effects on the nutrient digestibility.	[32]
Growing pigs	Dried full-fat TM larva meal	No ingredients were substituted (comparison with fishmeal-, meat meal- and poultry meal-based diets)	9.95%	8 (5 of adaptation)	Higher AIDC of the lysine, histidine, arginine and cysteine than the other protein sources. Higher SID of the GE and cysteine than the other protein sources.	[35]
Weaned piglets	Partially defatted HI larva meal	30% and 60% (soybean meal)	5% and 10%	61	No effects on the nutrient digestibility.	[33]
Weaned rabbits	HI larva fat	No ingredients were substituted (comparison with extruded linseed-based diets)	30% and 60%	70	No effects on the nutrient digestibility.	[39]
Weaned rabbits	HI and TM larva fat	50% and 100% (soybean oil)	0.75% and 1.5%	41	No effects on the nutrient digestibility.	[38]

Note: HI: *Hemetia illucens*; TM: *Tenebrio molitor*; MD: *Musca domestica*; SBM: soybean meal; AIDC: amino acid apparent ileal digestibility coefficients; DM: dry matter; CP: crude protein; EE: ether extract; GE: gross energy.

**Table 4:** Dietary insect meal inclusion and related impacts on growth performance of aquatic species in comparison with the control diet used in the different trials.

Species	Insect Species	Insect Form	% Substitution (Ingredient)	% Insect Inclusion	Days of Feeding	Impact on Growth Performance	Reference
Atlantic salmon	Defatted HI	Larva meal	33%, 66% and 100%(FM)	4.91%, 9.84% and 14.75%	114	The FI and daily growth increased, and FCR were unaffected by the inclusion of the HI meal in the diets.	[15]
			85% (CP)	60%	56	Small differences in the growth performance, and no effects on the FI or FCR.	[14]
Carp var Jian	Defatted HI	Larva meal	25%, 50%, 75% and 100% (FM)	3.5%,7%, 10.5% and14%	56	No differences for the growth performance.	[45]
Clownfish	Defatted HI	Larva meal	25%, 50% and 75% (FM)	20%,40% and 60%	106	No differences for the survival and growth performance.	[46]
European seabass	Defatted HI	Pre-pupa meal	15%,30% and 45% (FM)	6.5%, 13% and19.5%	62	No differences among the groups for the growth performance and feed utilization.	[17]
	Full-fat TM	Larva meal	25% and 50% (FM)	25% and 50%	70	The TM50% inclusion level showed the lowest FW, WG, SGR, and feeding rate.	[9]
Gilthead seabream	Full-fat TM	Larva meal	33.4% and 74% (FM)	25% and 50%	163	The TM25% had higher FW, SGR, WG%, PER, and lower FCR than the other diets.	[8]
Mandarin fish	Full-fat TM	Larva meal	10.76%, 21.5% and 32% (FM)	10%, 20% and 30%	56	The growth rates and efficiency of nutrient utilization increased up to 20% for TM levels and declined as dietary TM level increased from 20 to 30%.	[40]
Pacific white shrimp	Full-fat TM	Larva meal	25%, 50%, 75% and 100% (FM)	7.6%, 15.3%, 22.9% and 30.5%	42	The WG, SGR, FI, feed conversion, and survival were not affected.	[11]
	Partially defatted HI	Larva meal	20%, 40%, 60%, 80% and 100% (FM)	7.1%, 14.1%, 21.2%, 28.3% and 36.3%	63	The FW, WG, SGR, and FCR were not modified up to a 25% FM dietary replacement.	[47]
Pearl Gentian grouper	Defatted TM	Larva meal	6.25%, 12.5%, 18.75%, 25% and 31.25% (FM)	2.5%, 5%, 7.5%, 10% and 12.5%	50	Lower FW and WG rate in 6.25 TM% diet than the other diets. The FCR of fish fed on 12.5 and 18.75 TM% showed no significant differences when compared to the control diet.	[42]
Rainbow trout	Partially defatted HI	Larva meal	25%, 50% and 100% (FM)	6.6%, 13.2% and 26.4%	84	The growth performance and feed utilization were positive and acceptable at all inclusion levels.	[16]
			25% and 50% (FM)	20% and 40%	78	The survival, growth performance and FCR were not affected	[18]
	Fish offal-enriched HI	Pre-pupa meal	25% and 50% (FM)	16.4% and 32.8% (N); 18.12% and 36.24% (E)	56	No significant differences for the growth performance.	[44]
	Partially defatted HI	Larva meal	46% (FM)	28.1%	49	No significant differences for the growth performance.	[43]
Rockfish	Full-fat TM	Larva meal	9.6, 19.9, 28.6, 38.1% (FM)	8%,16%,24% and 32%	56	The WG and SGR increased with increasing dietary inclusion of TM from 0 to 16% and then tended to decrease with further increase in dietary TM levels to 32%.	[41]

Note: HI: *Hemetia illucens*; TM: *Tenebrio molitor*; FM: fish meal; FW: final weight; FI: feed intake; FCR: feed conversion ratio; PER: protein efficiency ratio; SGR: specific growth rate; WG: weight gain.

**Table 5:** Dietary insect inclusion and related impacts on growth performance of poultry in comparison with the control diet used in the different trials.

Avian Species	Insect Species	Insect Form	% Substitution (Ingredient)	% Insect Inclusion	Days of Feeding	Impact on Growth Performance	Reference
Broiler chickens	HI	Larva meal	No specific ingredients were substituted (comparison with corn and SBM-based diets)	5%, 10% and 15%	35	No influence on the growth performance.	[22]
		Larva meal	No specific ingredients were substituted (comparison with maize grain, wheat pollard, fish meal and SBM- based diets)	5%, 10% and 15%	49	No influence on the growth performance.	[50]
	Defatted HI	Larva meal	No specific ingredients were substituted (comparison with corn, gluten and SBM- based diets)	5%, 10% and 15%	35	Improved final LW and FI during the starter period up to 10% of inclusion.	[51]
	TM	Larva meal	No specific ingredients were substituted (comparison with sorghum- SBM- based diets)	5% and10%	15	No influence on the growth performance.	[59]
		Larva meal	No specific ingredients were substituted (comparison with corn meal, gluten and SBM - based diets)	5%, 10% and 15%	40	Improved LW and FI with increasing levels of TM meal inclusion, but the feed efficiency resulted partially impaired.	[61,62]
		Larva meal	100% (SBM)	29.65%	32	Improved FCR.	[26,63]
		Not specified	0.5%, 1%, 2% and 10% of basal diet	NS	35	Improved final LW, FI and FCR at inclusion level of 1 % TM.	[65]
	BM MD TM	Larva meal	100% (SBM)	7.8% (BM) 8.0% (MD) 8.1% (TM)	35	Reduced feed consumption and FCR. Increase in the BWG.	[64]
	MD	Maggot meal	20%, 50%,75% and 100% (groundnut cake)	5.5%, 11%, 16.5% and 22%	42	No influence on the growth performance.	[66]
		Maggot meal	50% and 100%	2.25%, 4.50% and 6.75%		Increase in the LW without influence on the FCR.	[67]
		Maggot meal	No specific ingredients were substituted (comparison with corn meal, gluten meal and SBM-based diets)	5%, 10%, 15% and 20%	35	Increase in the BWG in the 10% and 15% groups.	[29]
		Maggot meal	20%,, 30%, 40% and 50% (FM)	0.80%, 1.20%, 1.60% and 2%	35	Increase in the FCR.	[68]
	HI	at	50% and 100%	3.43% and 6.87%	36	No influence on the growth performance.	[71]
	TM	Oil	5% of the basal diet	-	28	Decrease in the FI and FCR, but no effect on the BWG.	[31]
Free-range chickens	TM	Larva meal	100% (corn gluten meal)	7.5%	54	No influence on the growth performance.	[60]
Slow-growing organic broiler	HI	Larva meal	50% (SBM)	7.8%	75	No influence on the growth performance.	[49]
Quails	HI	Larva meal	No specific ingredients were substituted (comparison with SBM and whole wheat-based diets)	10% and 15%	28	No influence on the growth performance.	[24]
Quails female Japanese	HI	Larva meal	No specific ingredients were substituted (comparison with corn meal, SBM, rice bran meal and FM-based diets)	25%, 50%, 75% and 100%	140	Affected FI, FCR and egg weight, but not affected egg production.	[58]
Barbary partridges	HI	Larva meal	10% and 19%	25, 50%	64	Increase in the LW with better FCR than the control diet.	[52]
Broiler ducks	Defatted HI	Larva meal	33.6%, 66.6% and 100% (gluten meal)	3%, 6% and 9%	47	No influence on the FI and FCR.	[30]
Laying hens	HI	Larva meal	No specific ingredients were substituted (comparison with soybean cake, corn, wheat)	50% and 100%	21	No influence on the growth performance.	[53]
		Larva meal	-	0.5% and 1%	-	No influence on the growth performance.	[54]
		Larva meal	100% (SBM)	72.34%	147	More favorable FCR, but lower lay percentage, FI, average egg, laying hens weight and egg mass, than the control diet.	[55]
		Larva meal	No specific ingredients were substituted (comparison with SBM and maize grain-based diets)	25% and 50%	140	No influence on the growth performance.	[27]
		Larva meal	39%	3.5%, 5% and 6.5%	112	Affected egg mass and lay percentage. Increase in the egg production and weight.	[57]
		Larva meal		7.5%	56	Increase in the FI and FCR.	[56]
	MD	Maggot meal	No specific ingredients were substituted (comparison with cassavaroot meal and FM-based diets)	6.25%, 12.5%, 18.7% and 25%	56	No influence on the growth performance.	[69]
		Live larvae		3% and 5%	420	No influence on the FI. Improvement of the clutch size, egg weight, number of eggs hatched and chick weight.	[70]

Note: HI: *Hemetia illucens*; TM: *Tenebrio molitor*; BM: *Bombyx mori*; MD: *Musca domestica*; SBM: soybean meal; FM: fish meal; LW: live weight; BWG: body weight gain; FI: feed intake; FCR: feed conversion ratio; NS: not specified.

**Table 6:** Effects of dietary insect productsinclusion on growth performance of pigs and rabbits in comparison with the control diet used in the different trials.

Animal Species	Insect Species	% Substitution (Ingredient)	% Insect Inclusion	Days of Feeding	Impact on Growth Performance	Reference
Sows and off springs	MD larva meal	NS	NS	NS	No effects on the growth performance.	[75]
Weaning pigs	MD larva meal	NS	NS	NS	Improvement of the WG.	[78]
Weaning pigs	MD larva meal	NS	10%	NS	No effects on the growth performance.	[76]
Weaner pigs	MD larva meal	100% (FM)	10.8%	10 weeks	No effects on the growth performance.	[79]
Weaning pigs	BM larva meal	NS	NS	NS	Improvement of the WG.	[79]
Early weaned piglets	Dried MD larva meal in association with rumen content (mixture)	25%, 50%, 75% and 100% (wheat offal)	5%, 10%, 15% and 20%	17 weeks	Lower FI than the control diet and other MD-based diets at 20% level of MD larva meal inclusion.	[37]
Weaning pigs	TM protein concentrate	NS	Up to 6%	NS	Linear improvement of the body weight and body weight gain with increasing levels of dietary TM inclusion.	[74]
Weaning pigs	Full-fat HI larva meal	65% (full-fat SBM)	3.5%	27	No effects on the growth performance.	[72]
Weaning pigs	Dried full-fat TM larva meal	5%, 10%, 15% and 20% (SBM)	1.5%, 3.0%, 4.5% and 6.0%	35	Linear increase of the BW, ADG and ADFI with increasing levels of dietary TM inclusion (phase I).	[34]
Weaned piglets	Partially defatted HI larva meal	75% (SBM)	21%	21	No effects on the growth performance.	[73]
Weaned piglets	Full-fat and defatted pre-pupa meals	50% and 100% (full-fat HI prepupa meal replacing toasted soybeans); 70% (defatted HI prepupa meal replacing toasted soybeans)	4% and8% (full-fat HI prepupa meal); 5.4% (defatted HI prepupa meal)	15	No effects on the growth performance.	[32]
Weaned piglets	Partially defatted HI larva meal	30% and 60% (SBM)	5% and10%	61	No effects on the growth performance.	[33]
Growing rabbits	BM larva meal	NS	NS	NS	No effects on the growth performance.	[80]
Weaned rabbits	HI larva fat	No ingredients were substituted	30% and 60%	10 weeks	No effects on the growth performance.	[39]
Weaned rabbits	HI and TM larva fat	50% and 100% (soybean oil)	0.75% and 1.5%	41	No effects on the growth performance.	[38]

Note: HI: *Hemetia illucens*; TM: *Tenebrio molitor*; BM: *Bombyx mori;* MD: *Musca domestica*; FM: fishmeal; SBM: soybean meal; BW: body weight; ADG: average daily gain; ADFI: average daily feed intake; FI: feed intake; WG: weight gain; NS: not specified.

**Table 7:** Effects of dietary insect meal inclusion on aquatic species quality traits in comparison with the control diet used in the different trials.

Aquatic Species	Insect Species	Insect Form	% FM Substitution	% Insect Inclusion	Days of Feeding	Impact on Flesh Quality (Raw/Cooked Fillets/Frozen)	Reference
Atlantic salmon	HI	Partially defatted larva meal	25% and 100%	5% and 25%	105	No differences for odour, flavour/taste or texture among the groups (cooked).	[84]
Carp var. Jian	HI	Partially defatted	25%, 50%, 75% and 100%	3.5%, 7%, 10.5% and 14%	56	No differences in the proximate composition, while the HI inclusion decreased the PUFA n3 content.	[45]
		Partially defatted larva meal	25% and 50%	20% and 40%	78	Significant changes in the perceived intensity of aroma, flavor and texture. Dominance of a metallic flavor characterized the fillets of fish fed HI diets (cooked).	[89]
Rainbow trout	HI	Partially defatted larva meal	25% and 50%	20% and 40%	78	50% inclusion: increased the EEcontent and induced a decrease of the valuable PUFA, with a worsening of the lipid health indexes (raw).	[18]
		Partially defatted larva meal	25% and 50%	20% and 40%	78	50% HI inclusion: decreased the fillet yellowness. Increase in the SFA (C12:0) and MUFA contents. Decrease in the PUFA content t(raw).	[86]
		Partially defatted larva meal	25% and 50%	20% and 40%	78	Differences in quality after 30 days of storage (frozen). No HIeffect on the pH, shear stress, color and water holding capacity of the fillets (frozen/cooked). HI50: increased the SFA and decreased the MUFA and the PUFA contents (frozen/cooked).	[88]
		Partially defatted larva meal	46%	28.1%	49	No differences in the chemical composition. Decrease in the PUFA (EPA and DHA) content. Increase in the SFA (C12:0) content. Slightly darker coloration of fish fed HI *vs* control. No organoleptic differences (cooked).	[43]
		Full-fat pre-pupa normal (N) or enriched (E)	25% and 50%	16.4% and 32.8% (N); 18.12% and 36.24% (E)	56	Sensory analysis: no significant difference for both the N and the E HI. Improvement of the EDA and DHA contents in the enriched HI fish (raw).	[44]
Rainbow trout	TM	Full-fat larva meal	25% and 50%	25% and 50%	90	Increase in the CP content and decrease in the EPA and the DHA contents (raw).	[10]
		Full-fat larva meal	25% and 50%	25% and 50%	90	No influence on the pH, water holding capacity, cooking loss, shear force and color (raw &cooked). No differences in the proximate composition. Decrease in the PUFA content (EPA and DHA).	[82]
Gilthead seabream	TM	Full-fat larva meal	33.4% and 74%	25% and 50%	163	No negative effect on the marketable indexes with a 25% of TM inclusion level. At 50% of TM inclusion level, dressed yield was penalized (raw).	[8]
Blackspot seabream	TM	Full-fat larva meal	26% and 51%	21% and 40%	131	TM50 *vs* FM fish fillets (fresh): higher yellowness and chroma, but lower hue. No effect on the proximate composition. Decrease in the PUFA content (EPA and DHA) and worsening in the lipid health indexes.	[81]
Pacific white shrimp	TM	Full-fat TM larva meal	25%, 50%, 75% and 100%	7.63%, 15.25%, 22.88% and 30.5%	42	Increase in the CL content. Decrease in the EPA and DHA contents. No differences in the proximate composition, color and firmness.	[85]
	HI	Partially defatted larva meal	20%, 40%, 60%, 80% and 100%	7.1%, 14.1%, 21.2%, 28.3% and 36.3%	63	Maximum whole-body CP and CL content achieved at 29% and 15% of HI inclusion, respectively.	[47]

Note: HI: *Hermetia illucens*; TM: *Tenebrio molitor*; FM: fish meal; EE: ether extract; SFA: saturated fatty acids; MUFA: monounsaturated fatty acids; PUFA: poly unsaturated fatty acids;DHA: Docosahexaenoic acid; EPA: Eicosapentaenoic acid; CP: crude protein; CL: crude lipid.

**Table 8:** Effects of dietary insect meal inclusion on egg and meat quality traits in comparison with the control diet used in the different trials.

Avian Species	Insect Species	Insect Form	% of Substitution (Ingredient)	% Insect Inclusion	Days of Feeding	Impact on Carcass Characteristics and Meat Quality	Reference
Broiler chickens	HI	Larva meal	100% (SBM)	29.65%	32	No influence on the carcass characteristics and broiler meat color.	[26]
		Larva meal	50% (SBM)	7.8%	75	No influence on the carcass characteristics. Cooking loss increased with the HI plus pea protein diet compared with the control.	[49]
		Larva meal	No specific ingredientswere substituted (comparison with maize grain, wheat pollard, FM and SBM- based diets)	5%, 10% and 15%	49	No influence on the carcass characteristics and sensory properties (taste and aroma) of the cooked breast meat.	[50]
		Larva meal	50% (SBM)	11.9% and 14.5%	34	HI meal results in a product that does not differ from the standard fed control group, with the exception that the breast filet has a more intense flavor that decreases over storage time.	[93]
		Pre-pupa meal	NS	5%, 10% and 15%	32	No influence on the carcass characteristics. No significant differences for the pH, color, thaw loss and cooking loss as well on the sensory characteristics (aroma, flavor, juiciness, and tenderness) of the breast muscle of the broilers fed HI meal.	[90]
		Larva meal	No specific ingredientswere substituted (comparison with corn, gluten and SBM- based diets)	5%, 10 % and 15%	35	Higher live and carcass weight up to 10% than the other groups. Lower yellowness values in HI groups than the control diet. Higher CP and lower moisture contents than the other diets. Higher percentages of C12:0, C14:0 and C16:0 but lower PUFA rates than the control diet.	[91]
	TM	Larva meal	No specific ingredientswere substituted (comparison with corn meal, gluten and SBM- based diets)	5%,10% and 15%	40	Increase in the carcass weight, abdominal fat weight and percentages.	[61]
		Larva meal	No specific ingredientswere substituted (comparison with corn meal, gluten and SBM- based diets)	5%,10% and 15%	40	No influence on the carcass characteristics.	[62]
		Larva meal	100% (SBM)	NS	32	Higher percentages of C12:0 and C14:0 in the intramuscular fat of broilers fed the TM larva meal diet than the control diet.	[96]
		Not specified	0.5%, 1%, 2% and 10%	NS	35	Improvement inthe slaughter yield, dressed carcass and eviscerated weights in birds fed TM diets at 2% inclusion level. High TM levels affect the CL content.	[65]
	MD	Larva meal	100% (Fish meal)	10%	32	Higher live and carcass weights, and breast and thigh yield than the control diet. Meat quality parameters were not affected except for the redness and drip loss that were the lowest in the HI meal-treated group. Higher metallic aroma and aftertaste, and sustained juiciness values were found in the MD larva-fed chickens than the control group.	[92]
		Maggot meal	50%, 75 % and 100%	2.25%, 4.50% and 6.75%	49	No significant difference on the hot carcass yield or proportion of different parts of the carcass, but higher proportion of the liver and gizzard than the control group.	[67]
	BM MD TM	Larva meal	100% (SBM)	7.8% (BM) 8.0% (MD) 8.1% (TM)	35	Tenderness and juiciness of meat were higher in the TM group compared to the control and other treatments.	[64]
	HI	Fat	50% and 100%	3.45% and 6.9%	35	No influence on the carcass characteristics and chemical and physical meat quality parameters. Increase in the SFA content to the detriment of the PUFA fraction in the breast meat of the HI fat groups.	[99]
	TM	Oil	5% of the basal diet	NS	28	Positive effect on the SFA, MUFA and PUFA contents, but negative effect on the AI and TI values.	[31]
Barbary partridges	HI TM	Larva meal	10% and 19%	25% and 50%	64	No effect on the raw meat color, but increase in the yellowness index of the cooked meat was observed. No differences in the chemical composition of the raw and cooked meat. Higher SFA and MUFA rates and lower PUFA rates than the control diet.	[94]
	HI TM	Larva meal	10% and 19%	25% and 50%	55	The carcass weights of all the insect groups were higher than the SBM group.	[52]
Quail	HI	Larva meal	24.8% (SBM)	10% and 15%	28	Breast meat weight and yield did not differ, while the inclusion of HI meal affected the meat pH and redness value. Meat proximate composition, cholesterol content and oxidative status remained unaffected by HI supplementation, as well as its sensory characteristics and off-flavors perception. Increase in the SFA content and decrease in the PUFA rates in the breast meat of the HI-fed quails.	[24,95]
Laying hens	HI	Larva meal	NS	0.5% and 1%	NS	A significant improvement of the appearance, texture and the taste and odor of eggs.	[54]
		Larva meal	100% (SBM)	17%	147	Hens fed the insect-based diet (HI) produced eggs with a higher proportion of yolk than the group fed the SBM group. HIM was associated with redder yolks, richer in γ-tocopherol, lutein, β-carotene and total carotenoids than SBM yolks.	[97]
		Larva meal	41% (SBM)	5% and 7.5%	182	Hens fed the HI-based diet linearly increased the yolk color, egg shell-breaking strength and egg thickness.	[56]
Free range laying hens	HI	Live larvae			42	Decrease in the egg weight, shell weight and thickness, and yolk color.	[98]

Note: HI: *Hemetia illucens*; TM: *Tenebrio molitor*; BM: *Bombyx mori*; MD: *Musca domestica*; EE: ether extract; CL: crude lipid; SFA: saturated fatty acids; MUFA: monounsaturated fatty acids; PUFA: polyunsaturated fatty acids: AI: atherogenicity index; TI: thrombogenicity index; SBM: soybean meal; FM: fish meal; NS: not specified.

**Table 9:** Effects of dietary insect products inclusion on meat quality of pigs and rabbits in comparison with the control diet used in the different trials.

Animal Species	Insect Species Tested	% Substitution (Ingredient)	% IM Inclusion	Days of Feeding	Impact on Meat Quality	Reference
Sows and off springs	MD larva meal	NS	NS	NS	More carcass meat with better organoleptic properties than the control diet.	[75]
Weaner pigs	MD larva meal	100% (FM)	10.8%	70	Higher slaughter weight and fat measurements than the control diet.	[77]
Weaned rabbits	Grasshopper meal	25%, 50%, 75% and 100% (FM)	1.25%, 2.50%, 3.75% and 5%	63	Higher slaughter and carcass weights than the control diet and the other grasshopper meal-based diets at 2.50% level of grasshopper meal inclusion. Higher dressing percentage than the control diet and the other grasshopper meal-based diets at 1.25% level of grasshopper meal inclusion. Higher abdominal fat percentage than the control and 5% grasshopper meal-based diets at 1.25, 2.50 and 3.75% level of grasshopper meal inclusion.	[101]
Weaned rabbits	HI larva fat	No ingredients were substituted	30-60%	70	Lower FA content, higher 12:0 and 14:0 contents, and worst atherogenic index, thrombogenic index and n-3/n-6 ratio than the extruded linseed-based diets.	[100]

Note: IM: insect meal; MD: *Musca domestica*; HI: *Hemetia illucens*; FM: fishmeal; FA: fatty acids; NS: not specified.
